# Fast vehicle detection based on colored point cloud with bird’s eye view representation

**DOI:** 10.1038/s41598-023-34479-z

**Published:** 2023-05-08

**Authors:** Lele Wang, Yingping Huang

**Affiliations:** School of Optical-Electrical and Computer Engineering, University of Shanghai for Science and Technology, Shanghai, 200093 China

**Keywords:** Electrical and electronic engineering, Engineering

## Abstract

RGB cameras and LiDAR are crucial sensors for autonomous vehicles that provide complementary information for accurate detection. Recent early-level fusion-based approaches, flourishing LiDAR data with camera features, may not accomplish promising performance ascribable to the immense difference between two modalities. This paper presents a simple and effective vehicle detection method based on an early-fusion strategy, unified 2D BEV grids, and feature fusion. The proposed method first eliminates many null point clouds through cor-calibration. It augments point cloud data by color information to generate 7D colored point cloud, and unifies augmented data into 2D BEV grids. The colored BEV maps can then be fed to any 2D convolution network. A peculiar Feature Fusion (2F) detection module is utilized to extract multiple scale features from BEV images. Experiments on the KITTI public benchmark and Nuscenes dataset show that fusing RGB image with point cloud rather than raw point cloud can lead to better detection accuracy. Besides, the inference time of the proposed method reaches 0.05 s/frame thanks to its simple and compact architecture.

## Introduction

Object detection is a prerequisite for 3D scene perception^1^ by autonomous driving, which has drawn significant attention from academia and industry. The purpose is to perceive the position and size of objects with a bounding box in the real world.

Autonomous vehicles are commonly available with diverse sensors, among which RGB cameras and 3D LiDAR sensors can provide information for accurate detection. For example, RGB images acquired by camera are enriched with texture, color, and high resolution. As passively operated sensors, cameras are susceptible to varying lighting conditions and lack depth of objects. LiDARs provide more accurate and farther distance measurements in the physical world. However, the irregular point cloud usually becomes more sparse and unstable as the distance from scanner center increases, which the relationships are insufficient to extract geometric features. It not only brings a challenging design of rational convolution model, but also demands further thoughts on data representation.

According to the sensors used by vehicle detection methods, recent studies with deep learning can be separated into two mainstream categories: LiDAR-based and multi-modal fusion-based approaches. The point cloud data collected by LiDAR is characterized by disorder and sparsity, which first needs to be processed structurally. Previous LiDAR-based methods either convert point clouds into 2D projected views^2–11^ or voxel grids^12–18^ by using Convolutional Neural Networks, or directly take raw point clouds using PointNet^19–21^.

The existing multi-modal fusion-based approaches can be divided into three categories. First, early level-fusion^22–24^, refers to integrating the sensor data directly, such as augmenting LiDAR point clouds with corresponding semantic labels^22^. Second, middle-level fusion^25–39^, first acquires 2D projected views (bird’s eye view and front view), and then extracts view features independently^25,26,28,29^. After that, features from multiple sensor data are directly concatenating at the proposal network, which may weaken consistency features. The typical methods^38,39^ apply “continuous feature fusion” to enable the sharing of feature information on all strides of image and point cloud networks. One subtle but important drawback is “feature blurring”. This happens because each feature vector from the BEV matches to multiple pixels in the image view, and vice versa. Some existing middle-level fusion methods are limited by cascade processing^37^, utilizing diverse sensors at different phases. For instance, RGB images are utilized in the first phrase and point clouds are applied in the second phrase. Though the cascade method uses multi-modal data, when the shortcoming of one sensor still exists, the other sensor cannot be eliminated. The third is late-level fusion^40–42^, it processes each modality on a separate network and incorporates 2D and 3D results based on interrelationships or special models. This strategy has much reliance on the results of 2D tasks, and involves less interaction between different modalities.

Above all, these middle-level and late-level fusion strategies must run two deep learning approaches to extract specific-views features, the detection performance is restricted by the decision results. Compared to late-level fusion, middle-level fusion-based methods utilize a complicated pipeline to address the various modalities sequentially, which are generally computationally inefficient, making integration into an autonomous system challenging. Since the early-level fusion strategy registers two modalities into a unified coordinate system, which can achieve fusion of limited perception points. Then the perception points are fed into the detection network. This can guide to a simpler design and increase the efficiency of convolution coding. The early-level fusion has advantages of fewer data and high potential for improvements in real-time. Thus, strategies based on early-level fusion need further research.

Different from Contfuse^38^ and lecture^39^, which both belong to middle-level fusion. The former designs an end-to-end learnable architecture, based on KNN, bi-linear interpolation, and a learned continuous convolution to merge image and LiDAR feature maps at various resolution levels. However, interpolation process creates a smoothness effect that needs deeper and heavier DCNN to remedy. The latter proposes a sparse non-homogeneous pooling layer to transform features between bird’s eye view and front view. The feature ambiguity may occur at the fusion stage.

Our proposed method belongs to an early-level fusion strategy at the data level, to avoid these issues, it projects image pixels onto point cloud directly, augments 3D point with texture information to generate colored point clouds, and then utilizes a priori knowledge and spatial ensemble constraints to acquire 6-channel colored BEV maps.

Due to the disorderly and sparse nature of 3D data, for decades, the most commonly used point cloud processing method for real-time detection is bird's-eye view projection, which has many advantages. It can retain the relative position information of the object in 3D space. There is no occlusion and scale ambiguity, easy to generate feature points, and has better object location capability. However, while point cloud can trivially be turned to bird’s eye view, doing so is much more complicated for image. Hence, the core challenge in designing multi-modal fusion network lies in consolidating the LiDAR BEV map with RGB image.

The representative encoding–decoding method to hypothesize the location is Feature Pyramid Network (FPN) network. The features of different scales with two-row path are connected at the same scale, thus the final obtained features map contain different solutions, which can better detect objects with different sizes.

Note that for LiDAR detection methods operate directly on original 3D data, this method demands minimal network adaptations, such as changing the number of channels dedicated to reading the input.

Based on these issues, we present a simple and effective early-fusion based method for vehicle detection. The early fusion module projects the image pixels (RGB) into 3D space, and augments point cloud (XYZr) to generate the 7D colored sparse point cloud as the initial step. And a BEV encoding format is designed to unify the augmented data into the same representation as BEV maps. Then BEV maps are sent to a 2D Feature Fusion (2F) detection network to derive the semantic classes and bounding box. The proposed method is exclusively based on 2D CNN, without 3D convolution and other specialized manipulations, allowing an end-to-end optimization.

To summarize,the main contributions can be generalized:

1. This paper proposes an early fusion strategy to detect 3D vehicles from point clouds and RGB images, which has competitive accuracy and efficiency of detection.
2. To the best of our knowledge, this is the first method to project image pixels onto 3D space to generate 7D colored point clouds and converts them into BEV grid maps. And consider the colored BEV maps as multi-sensor fusion input.
3. The Feature Fusion(2F) detection model is designed to extract features.
4. Real-time execution with high accuracy is evaluated on the KITTI benchmark.

## Related work

In 3D scene perception, one of the primary challenges is that 3D data comprises a large field of view (FOV) of irregular and unorganized points, which not only requires in-depth consideration of data representation, but also needs to design the reasonable CNN architecture. Regarding the use of 3D LiDAR data for object detection in self-driving vehicles, various methods have been developed. This section introduces two mainstream methods, namely LiDAR-only and multi-modal data fusion.

### LiDAR-only-based detection

LiDAR-only based methods in terms of point cloud representations can be categorized into several groups, including projected 2D views^2–11^, voxel grids^12–18^, and pure point cloud-based methods^19^.

Projected 2D views-based methods: to decrease the calculation burden of 3D data, some previous algorithms have tried to combat sparsity by projecting 3D data into 2D images as pseudo images. This operation allows for an intensified and compressed representation of point cloud, which proves that a mature 2D detection framework can be applied for prediction. The VeloFCN^2^, LMNet^3^, and FVNet^4^ project point cloud as front view (FV), while BirdNet^5^, BirdNet+^6^, Complex-YOLO^7^, RT3D^8^, PIXOR^9^, PointPillars^10^ and HDNet^11^ as bird’s-eye-view (BEV) formats. Front view is similar to image and contains spatial coordinates. It is simpler to acquire the location and appearance. The BEV representation has further benefits than front view. For instance, it retains physical dimensions, directly provides the position on the ground surface, and the dimensions of the object do not modify due to distance. Moreover, the obfuscation issues of various objects occupying different spatial regions are solved. This projection solution has been intended to satisfy real-time requirements. Thus, the BEV representation has dominated the projection method.

Voxel grids-based methods: voxel-based approaches^12^ discrete 3D space into equal voxels and utilize standard 3D CNN^13,14^ to extract the feature of each voxel grid. Li^15^ proposes a framework to extend 2D FCN to 3D FCN for detection. The voxel-based feature extractor (VFE)^16^ has increased receptive field and added additional context to extract features. SECOND^17^ modified VoxelNet^16^ by adding sparse convolution to eliminate empty voxels, which enhanced the efficiency of 3D convolution. To better discriminate obscured vehicles, SegVoxelNet^18^ designs a depth-aware head endowed with different kernel sizes and convolutional layer expansion rates.

Pure point cloud-based methods: PointNet^19^ has been a promising method for straightly handling 3D data without any additional transformation or pre-processing. Based on the benefits of PointNet in terms of translation in-variance, local connections, and shared parameters, some specialized versions^20,21^ are subsequently launched. However, both computation capability and memory consumed to calculate 3D models increase cubically.

### Multi-modal data fusion-based detection

Multi-modal fusion-based approaches broadly follow three categories: early-level^22–24^, middle-level^25–34^, and late-level fusion^40,41^. Each is reviewed in turn.

Early-level fusion-based methods fuse the perception data in each modality directly by spatial alignment and projection at data stage, such as Pointpainting^22^, which utilizes DeeplabV3+ to yield per-pixel labels, and then projects these labels back to 3D space to obtain the decorated point clouds. It does not fuse the high-level features of the different modalities at all. PointAugmenting^24^ proposes to augment LiDAR points with the deep features extracted from the 2D image.

In Middle-level fusion-based methods^25–39^, the features are combined after feature extraction. MV3D^25^ and AVOD^26^ project point cloud into 2D projected views, then RGB images and 2D projected views are treated as inputs for different backbones, and then fuse the features from multiple views to predict bounding box. The two implements of BEV Fusion^31,32^ unify features from multi-modal inputs, which project image features onto shared BEV space and concatenate it with LiDAR feature. Transfusion^33^ relies on LiDAR BEV features and image guidance to generate object queries, and then utilizes attention mechanism to fuse these queries with image features. MSMDFusion^34^ designs multi-modal interaction in BEV space and voxel space to align spatial features from different sensors. Liu^35^ takes RGB image and two sparse depth maps as input, designs a spatial motion perception module to generate pseudo-LiDAR point cloud. Liang^36^ presents several tasks to assist object detection, which including 2D object detection, ground estimation, and depth completion. Gu^37^ proposes a cascade fusion strategy, the LiDAR data is sent to network to acquire sparse results in the first phrase, through sparse to dense module, the features of both modalities are merged in the fusion model.

Some methods attempt to merge two modalities by sharing the information across 2D and 3D backbones. The core problem is the feature mismatching between RGB image and point cloud. Wang et al.^39^ propose a sparse non-homogeneous pooling, and a projection matrix is utilized to transform features between RGB image and BEV. Contfuse^38^ utilizes a ResNet-18 to extract features on RGB image and point cloud BEV view separately, then performs interpolation of each BEV pixel position with RGB features based on K-nearest neighbor research, and last employees a parametric continuous convolution network to project them onto the BEV plane to merge with the BEV features. To utilize continuous convolutions, it requires the identification of K nearest 3D points for each grid, which is computationally expensive operation that may not meet real-time requirements as the density.

Late-level fusion-based methods incorporate 2D and 3D results from independent networks based on interrelationships or special models. F-PointNet^40^ and F-ConvNet^41^ utilize 2D detection to yield 2D proposals, which are re-projected to the 3D space, and then feed into a PointNet-like network to predict the 3D corresponding bounding box. Based on this methodology, framework^42^ applies YOLOV3 to obtain proposals, which are then cast onto frustums to yield highly accurate trajectories.

In contrast to existing methods that either utilize a complex pipeline to process different modalities or conduct late fusion, our simple yet effective fusion strategy allows the interaction between modalities to be learned at the early stage with a less computation-heavy network. We demonstrate that this approach is effective and has better detection results in distant and occluded objects, which can significantly improve detection performance.

## Method

**Figure 1:**
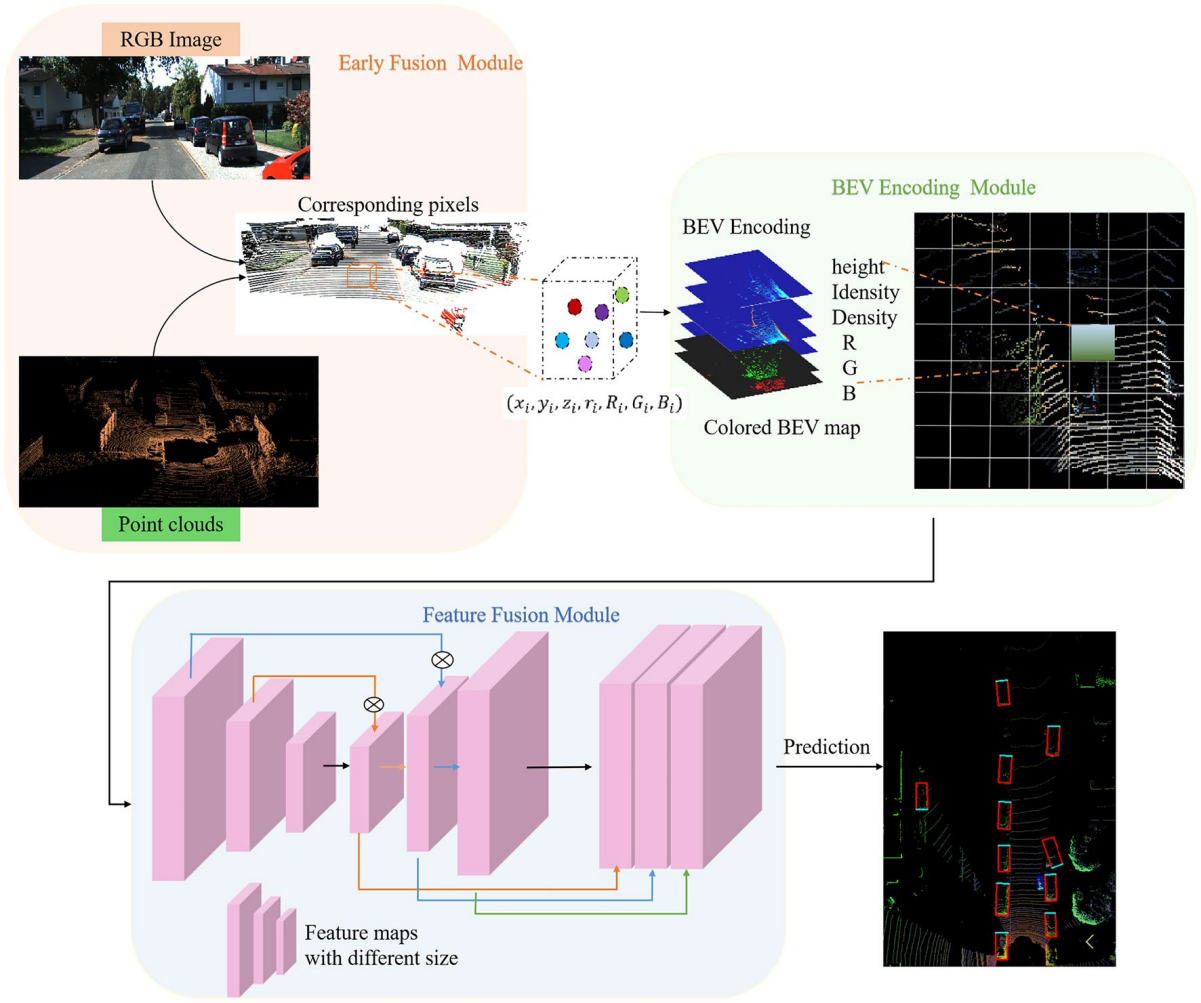
The proposed method architecture.

In this section, Fig. 1 gives an overview of the framework for 7D colored point cloud generation and proposed approach for vehicle detection in detail. It has two input modalities, comprising RGB images taken by the camera and sparse point cloud taken by Velodyne 64E LiDAR from KITTI^43^^.^

The main framework of this method comprises three modules. The first module is the Early fusion, which projects RGB image into the 3D space, augments point cloud data with color texture to generate 7D colored point cloud. The second module is BEV encoding format of 7D colored point clouds. It has been applied to unify 7D colored point cloud into 2D BEV grids and to convert point sets into feature vectors of uniform dimension. In the third module, BEV maps are fed into the Feature Fusion (2F) network to generate the proposals, and parameters are estimated from multi-layers of feature maps such as semantic class, boundary box, and orientation.

In this approach, the basic unit is 2D grids, it does not only reduce the point cloud’s dimension, but also better saves memory due to the input of smaller size. Furthermore, the RPN network in this model can utilize a deeper pyramid structure to capture rich features for improved performance.

### Early fusion module

Through synchronization and calibration parameters, two modalities have been calibrated. The transformation equation is:

1 $$\begin{array}{l}{P}_{cam}={R}_{rect}^{0}\cdot {T}_{velo}^{cam}{\cdot P}_{lidar}\end{array}$$

2 $$\begin{array}{l}{p}_{cam}={T}_{proj }\cdot {P}_{cam}\end{array}$$

3 $$\begin{array}{l}{T}_{velo}^{cam}=\left[\begin{array}{cc}{R}_{velo}^{cam}& {t}_{velo}^{cam}\\ 0& 1\end{array}\right]\end{array}$$

where ${R}_{rect}^{0}$ is the rotation matrix, ${T}_{velo}^{cam}$ is the transformation matrix and ${T}_{proj}$ is the projection matrix from camera coordinate systems.

In this way, image pixels are projected onto corresponding point data in 3D space according to projection matrix. Then the corresponding pixels (from RGB Camera) are assigned to the 3D data to generate 7D colored point cloud. Therefore, each obtained 7D colored point cloud not only contains 3D coordinates and the intensity of reflection, but also retains color and texture, which can be denoted as:${p}_{i}=\left({x}_{i},{y}_{i},{z}_{i},{r}_{i},{R}_{i},{G}_{i},{B}_{i}\right)$.

**Figure 2:**
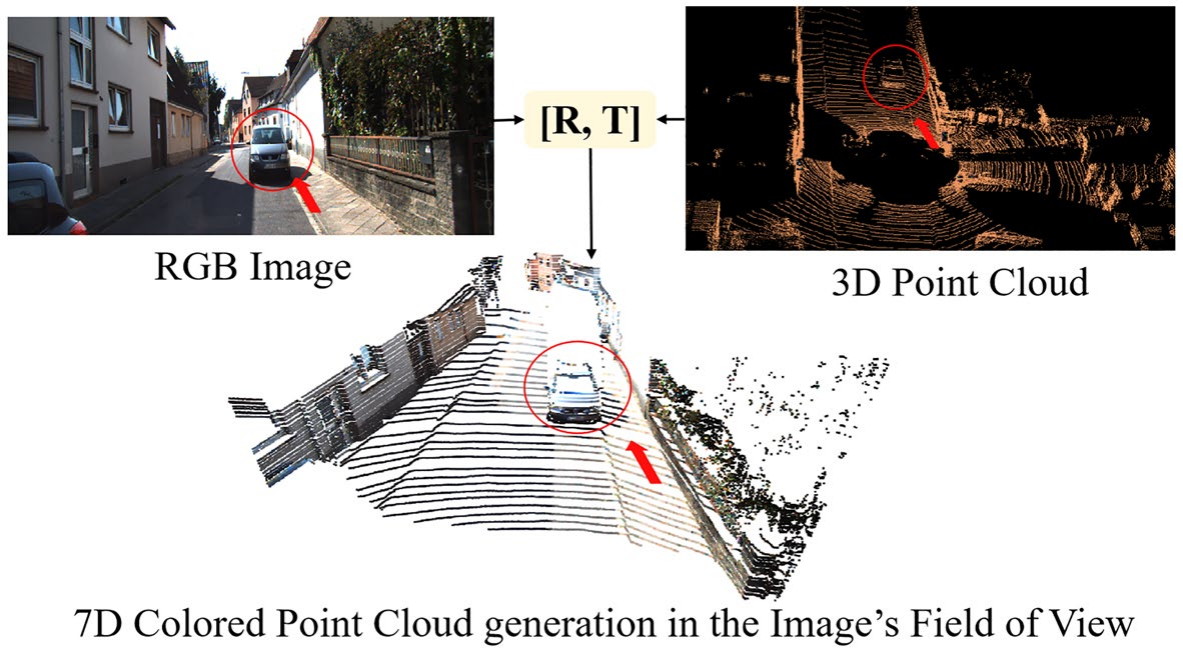
An illustration of 7D colored point cloud generation in the image's field of view. Through calibration matrix (calib.txt), image pixels are projected onto corresponding points. The $R$ and $T$ are the rotation and transformation matrix.

In order to achieve real-time availability and reduce unnecessary computation, detection range is set to $\left\{{\left[x,y,z\right]}^{{\varvec{T}}}| x\in [{0,70}]m, y\in [-{40,40}]m, z\in [-{3,3}]m\right\}$, discarding the remaining pixels. An illustration of 7D colored point cloud generation is illustrated in Fig. 2.

**Figure 3:**
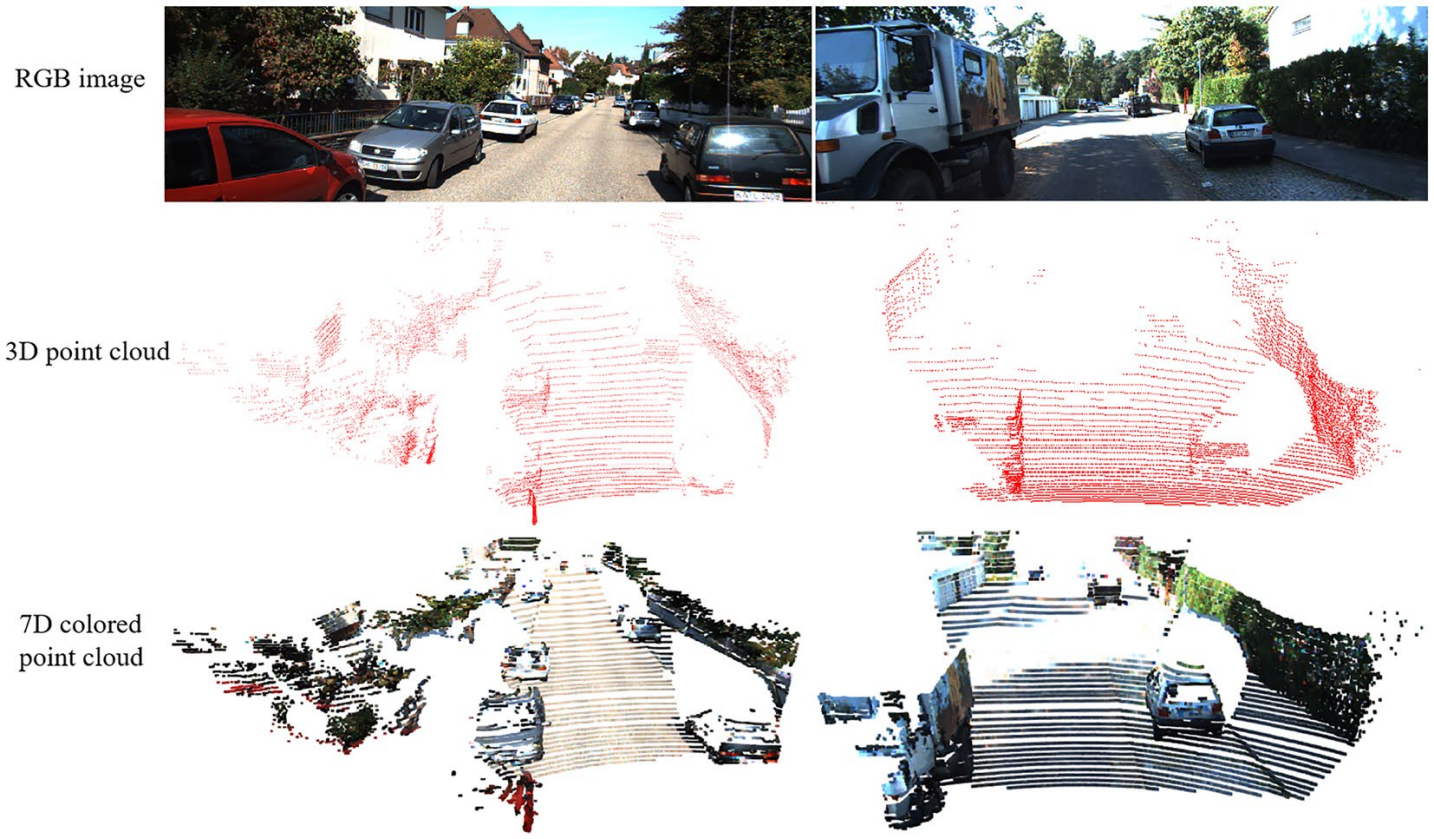
Some visual examples of RGB image, 3D point cloud and corresponding 7D colored point cloud.

Figure 3 represents an example of the different data on KITTI data set. The first row shows original RGB image, the second row shows 3D point cloud data within the image’s field of RGB images, and the third row is 7-dimensional colored point cloud. The image provides road environment information, and the 3D LiDAR data presents the object scanned by the sensor and its surrounding environment. The colored point cloud enhances the semantic information of 3D point cloud. Therefore, the 7D colored data constructed in this section not only retains the spatial characteristics of point clouds, but also enriches the semantic characteristics of surface points, which can avoid the dependence of feature extractors of point clouds on the shape of objects.

### BEV encoding

This stage uses a priori knowledge and spatial ensemble constraints to process the generated 7D colored point cloud, and then obtains the rich and compact 6-channel BEV maps, which can be considered as a pseudo image. There are two advantages. Firstly, the BEV grids allow each object to occupy an individual spatial position, which facilitates the reflection of relative positional relationships between objects and reduces the disturbance of overlap and occlusion. Secondly, 6-channel BEV maps can be processed directly by conventional convolution structures, implying less computation and faster detection.

The generated 7D colored point clouds are converted into the 2D grids and then converted into a 6-channel BEV map according to Eq. (4):

4 $$\begin{array}{l}feature=\left(\overline{H },\stackrel{-}{I, }\overline{D },\stackrel{-}{R,}\overline{G },\overline{B }\right)\end{array}$$

**Figure 4:**
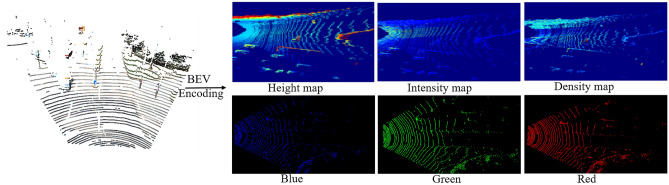
Schematic of 6-channel BEV maps generation. The 7D colored point cloud is converted into a pseudo-2D image composed of 6 channels.

In the above formula, $\overline{\mathrm{H} }$ is the average height, $\overline{\mathrm{I} }$ is the average intensity, $\overline{\mathrm{D} }$ is the average density, $\overline{\mathrm{R },} \overline{\mathrm{G} },\overline{\mathrm{B} }$ are the average primary colors, respectively. The conversion of generated 7D colored point cloud (left) and 6-channel colored BEV map (right) is illustrated in Fig. 4.

The specific conversion process is as follows: the obtained 7D colored point cloud is converted to $x-y$ plane, and the detection area is sliced into 2D grids with an interval of 0.1 m. Each grid covers an area of $0.1\mathrm{m }\times 0.1\mathrm{m}$ .

Step 1: The first channel is height map. The height feature is encoded as division of the maximum value $( \left \vert H\right \vert \mathrm{max})$ in each grid with the height in the detection region. The obtained normalized height value is encoded in the grid, i.e.:

5 $$\begin{array}{l}{H}_{i}=\frac{{max(H}_{Pro\,j}) -{H}_{min}}{{H}_{max}-{H}_{min}}\end{array}$$

where ${H}_{i}$ is the height value of the $i$ unit in the top view, ${H}_{Pro\,j}$ is the height value of the $j$ point, ${H}_{max}$ and ${H}_{min}$ are maximum and minimum values of height in the detection area, which are set to −3 and 3, respectively.

Step 2: The second channel is the intensity map. The intensity features are encoded as the average reflection intensity in each grid.

6 $$\begin{array}{l}{I}_{i}=\sum_{j=0}^{{n}_{i}}{I}_{Pro\,j}/{n}_{i}\end{array}$$

where ${I}_{i}$ is the intensity value of the $i$ grid in the top view, ${I}_{Pro\,j}$ is intensity value of $j$ th point, and ${n}_{i}$ is number of points falling on the $i$ th grid.

Step 3: The third channel is density map, which is encoded by the counts of points within each grid. In this case, the density is normalized by the Eq. (7):

7 $$\begin{array}{l}{D}_{i}={n}_{i}/ {n}_{max} \end{array}$$

where ${D}_{i}$ is the density of the $i$ th grid in the top view, and ${n}_{i}$ is the count of 3D points falling in the $i$ th grid. ${n}_{max}$ is the count of points in the grid with the highest density among all cells.

Step 4: The fourth to sixth channels are color features. The average value of color features in each grid is calculated respectively to get the average triplet $\overline{R }, \overline{G }, \overline{B }$.

In summary, the 7D colored point cloud is encoded as a 6-channel BEV image represented by the height, intensity, density of both two modalities data. On the one hand, it has a regular and structured format that can be easily processed, on the other hand, it is compact and does not require 3D convolution, saving computational resources.

### Feature fusion (2F) detection model

**Figure 5:**
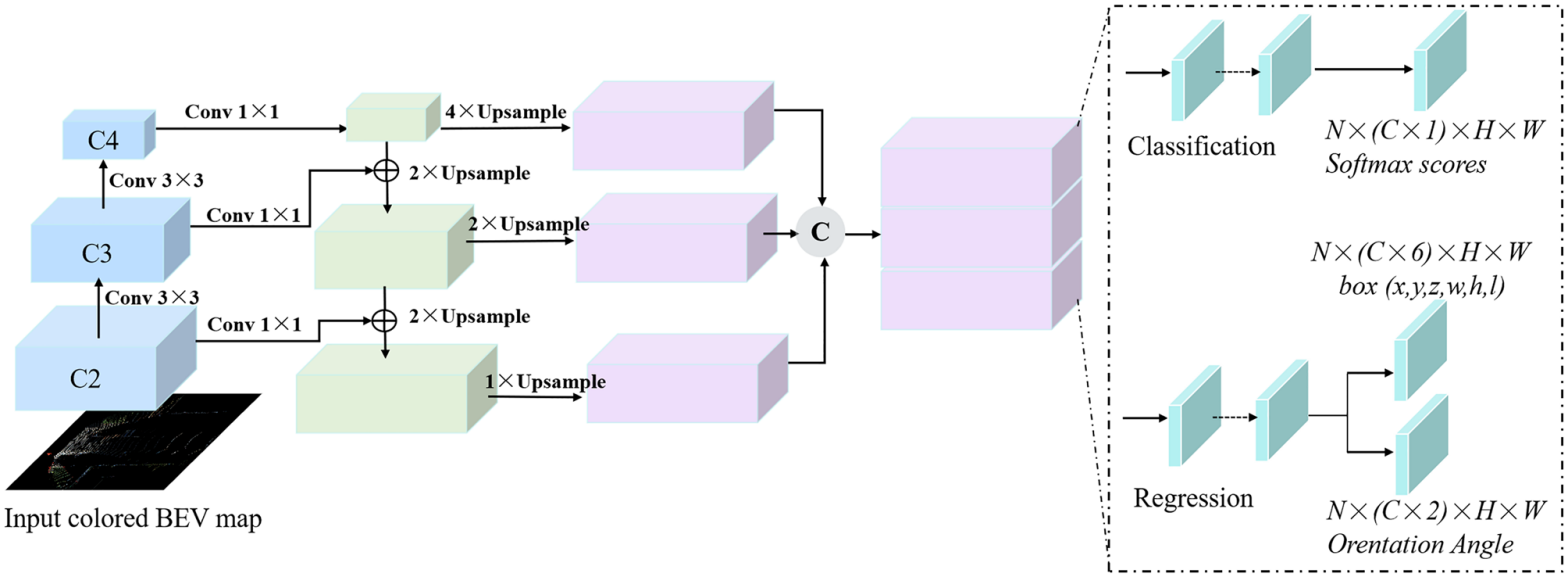
Feature fusion (2F) structure.

As illustrated in Fig. 5, the 2F model is basically an encoding–decoding framework, which applies ResNet-50^44^ in combination with a Feature Pyramid Network (FPN) structure^45^. Since the obtained colored BEV maps can provide rich information, which are utilized as input. To obtain accurate object location and semantics, the semantic texture of object is obtained by continuous down-sampling, and then the feature maps of high and low-level are combined to achieve multi-level feature fusion.

In *bottom to up* path, feature pyramids are constructed using the feature maps of C2, C3, and C4 of ResNet-50 at the scales of 1/2, 1/4, and 1/8.

In *up to bottom* path. After encoding, feature maps from each layer are passed to up-bottom, those encoded feature maps are then up-sampled several times on the account of the above responding layers to recover the input feature resolution, fusing by using 3 × 3 convolution and element average fusion operations. For the feature layer with the same original size, it can be regarded as the equivalent stage.

Due to position errors from several sampling operations, bottom-up high-level features are combined with low-level detailed features. Then these BEV feature map with strong semantics and high resolution can be obtained. Finally, the feature maps with multi-scale fusion can be obtained by concatenation, then they are passed to two Fully Connected layers to predict results.

Therefore, the generated feature pyramids are utilized to generate 2D proposals, with the multi-scale feature maps being provided by the multi-scale feature aggregation module.

### Loss function

The loss function for vehicle detection is similar to Pointpillars^10^ and SECOND^17^. It consists of three components: $smooth-{l}_{1}$ loss for position regression, ${L }_{cls}$ loss for object classification, and ${L}_{dir}$ loss for direction (heading Angle).

The parameters of the detection box are defined by $\left(x, y,z,w,l,h,\theta \right)$, where $x, y,z$ are center coordinates of the 3D box, $w,l,h$ are respectively width, length, and height, and $\theta$ is heading angle (object orientation). The parameters involved are as follows:

8 $$\begin{array}{l}\Delta x=\frac{{x}_{g}-{x}_{a}}{{d}_{a}}, \Delta y=\frac{{y}_{g}-{y}_{a}}{{d}_{a}}, \Delta z=\frac{{z}_{g}-{z}_{a }}{{d}_{a}}\end{array}$$

9 $$\begin{array}{l}\Delta l=log (\frac{{l}_{g}}{{l}_{a}}), \Delta h=log (\frac{{h}_{g}}{{h}_{a}}) \end{array}$$

10 $$\begin{array}{l}\Delta w=log (\frac{{w}_{g}}{{w}_{a}}), \Delta \theta ={\theta }_{g}-{\theta }_{a}\end{array}$$

In the above equation, $\Delta x$, $\Delta y$, $\Delta z$ are the offset between ground truth ${x}_{g}$ and predicted values ${x}_{a}$, normalized by the diagonal in the detection box: ${d}_{a}=\sqrt{{\left({l}_{a}\right)}^{2}-{\left({w}_{a}\right)}^{2}}$

- Regression position loss ${L}_{loc}$: the classification predictions are supervised with the cross entropy (CE) loss.
  11 $$\begin{array}{l}smooth-{l}_{1}\left(x\right)=\left\{\begin{array}{l}0.5{x}^{2}\left \vert x\right \vert <1\\ \left \vert x\right \vert -0.5,others\end{array}\right.\end{array}$$
- Object classification loss ${L}_{cls}$: in traffic scenes, the serious imbalance of positive and negative sample ratio is always an important factor affecting vehicle detection performance. Generally, the network generates approximately 70k boxes, there are only a few ground truth, each of which generates only 4–6 positives. This results in an extreme imbalance between foreground and background classes. Thus the focal loss is utilized to solve this problem.
  12 $$\begin{array}{l}{L}_{cls}={-\alpha \left(1-p\right)}^{\gamma }log\left(p\right)\end{array}$$
  where $p$ is the classification probability of predicted box, $\alpha$ is a weighted factor to balance strength of positive and negative examples, and $\gamma$ is focusing parameter, and $\alpha$ are $\gamma$ set to 0.25 and 2, respectively.
- Directional (heading angle) loss ${L}_{dir}$: since the angle has two directions $\left\{+,-\right\}$, and the angle regression loss can not distinguish the orientation. A softmax function is used to compute the discretized orientation loss. If the heading angle around Z-axis of the ground truth is greater than 0, the orientation is positive; otherwise, the orientation is negative.

By combining the losses discussed above, the overall loss function can be formulated as follows:

13 $$\begin{array}{l}L=\frac{1}{{N}_{pos}}\left({L}_{loc}{\beta }_{loc}+{L}_{cls}{\beta }_{cls}+{L}_{dir}{\beta }_{dir}\right)\end{array}$$

where ${N}_{pos}$ is the number of correctly detected boxes, ${\beta }_{loc}$,${\beta }_{cls}$ and ${\beta }_{dir}$ are weight of regression, classification, and direction, which are set to 2.0, 1.0, and 0.2, respectively.

## Experiments

### Dataset and metrics

This method conducts experiments on the KITTI^43^ object dataset and nuScenes^46^ to evaluate. KITTI object dataset consists of two parts: the training dataset with ground truth, containing 7481 samples, and the test dataset without ground truth, containing 7581 test samples. Eight categories are labeled, but only three categories–vehicle, pedestrian, and cyclists—are officially provided for evaluation. For each category quantitative evaluation, according to visible range of the object within the image's field of view, the data is classified into three difficulty levels, including Easy, Moderate, and Hard, with bounding box overlap (IOU threshold) of 70% for vehicle and 50% for other categories. According to the official evaluation, if the correct coincidence degree between the predicted detection box and the object reaches the threshold, the prediction can be considered correct.

The nuScenes dataset is much bigger than KITTI dataset. It has full annotations to support all sorts of tasks (3D object detection, tracking and BEV map segmentation).In this work, we utilize LiDAR point clouds and RGB images. 10 categories are evaluated: cars, trucks, buses, trailers, construction vehicles, pedestrians, motorcycles, bicycles,traffic cones and barriers.

### Implementation details

To verify the performance of the 3D object detection method on the basis of generated 7D colored point cloud. Training set is split into two non-overlapping subsets for training (3712) and validation (3769).

In the training part, the whole model uses Adaptive Moment Estimation (Adam) as the network optimizer to update model parameters. Momentum ranges from 0.95 to 0.85, the initial learning rate is 0.001, fixed-weight decay coefficient is 0.001, the batch size is 12, and the epoch is 300. The model is executed with the Pytorch 1.6 with NVidia 1080ti GPU. During model evaluation, only this method involves 7D colored point cloud as input, and the others utilize the original 3D point cloud.

## Experimental results

### Experimental comparison between 7D colored point cloud and 3D point cloud

To verify the effectiveness of the proposed fusion method with different combinations. Table 1 illustrates comparison of detection results of various input data on KITTI validation data. It uses 3D point cloud (first row) and 7D colored point cloud (second row) data as network input respectively. When using 3D point cloud, the input channel of network is changed to 3.

**Table 1:** Comparison results under different inputs on validation dataset (/%).

Input data	Vehicle			Pedestrian			Cyclists		
	E	M	H	E	M	H	E	M	H
3D point cloud	85.14	75.85	73.03	57.99	53.46	50.46	60.38	55.72	52.89
7D colored point cloud	86.99	76.26	74.32	61.04	57.08	52.82	63.98	57.25	53.52

The difficulty levels of "Easy (E)", "Medium (M)", and "Hard (H)" are defined by the KITTI official website. As can be observed in the three categories of vehicle, pedestrian, and cyclists, the detection results obtained by using both modalities as input are higher than only using 3D point cloud. When detecting pedestrians and cyclists categories, the accuracy is improved significantly, the reason is that the color information of the image makes input semantic information richer, and the multi-scale fusion feature network has certain advantages in detecting distant objects.

**Figure 6:**
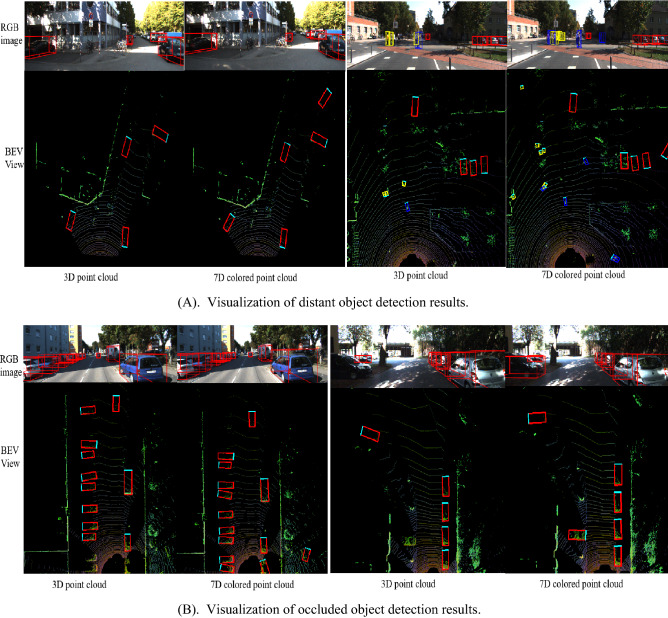
Detection visualization results of 3D point cloud and the generated 7D colored point clouds.

To visually compare the detection effects of two different data, Fig. 6 presents some visualization detection results of occluded and distant objects on KITTI validation set, from both image view and bird 's-eye view, respectively. Among them, in each sub-block, left column is the detection result that only takes 3D point cloud as input, and right shows visualization results using 7D colored point cloud as input. Figure 6A shows the distant object detection results, and Fig. 6B shows occluded object detection results. The red detection boxes represent vehicles, the blue detection boxes represent cyclists, and the yellow detection boxes represent pedestrians. The green part in the detection box indicates the orientation of the object.

It can be concluded that in a complex and crowded environment, when using sparse pure 3D point cloud, the situation of missing detection occurs owing to the absence of color information. In addition, with many surrounding obstacles and other interfering factors, the image can make up for the low resolution of LIDAR to a certain extent and make the detection more accurate. In conclusion, the detection network using multi-modal data has good robustness, which alleviates the problems for frequently encountered problems in traffic scenarios.

**Figure 7:**
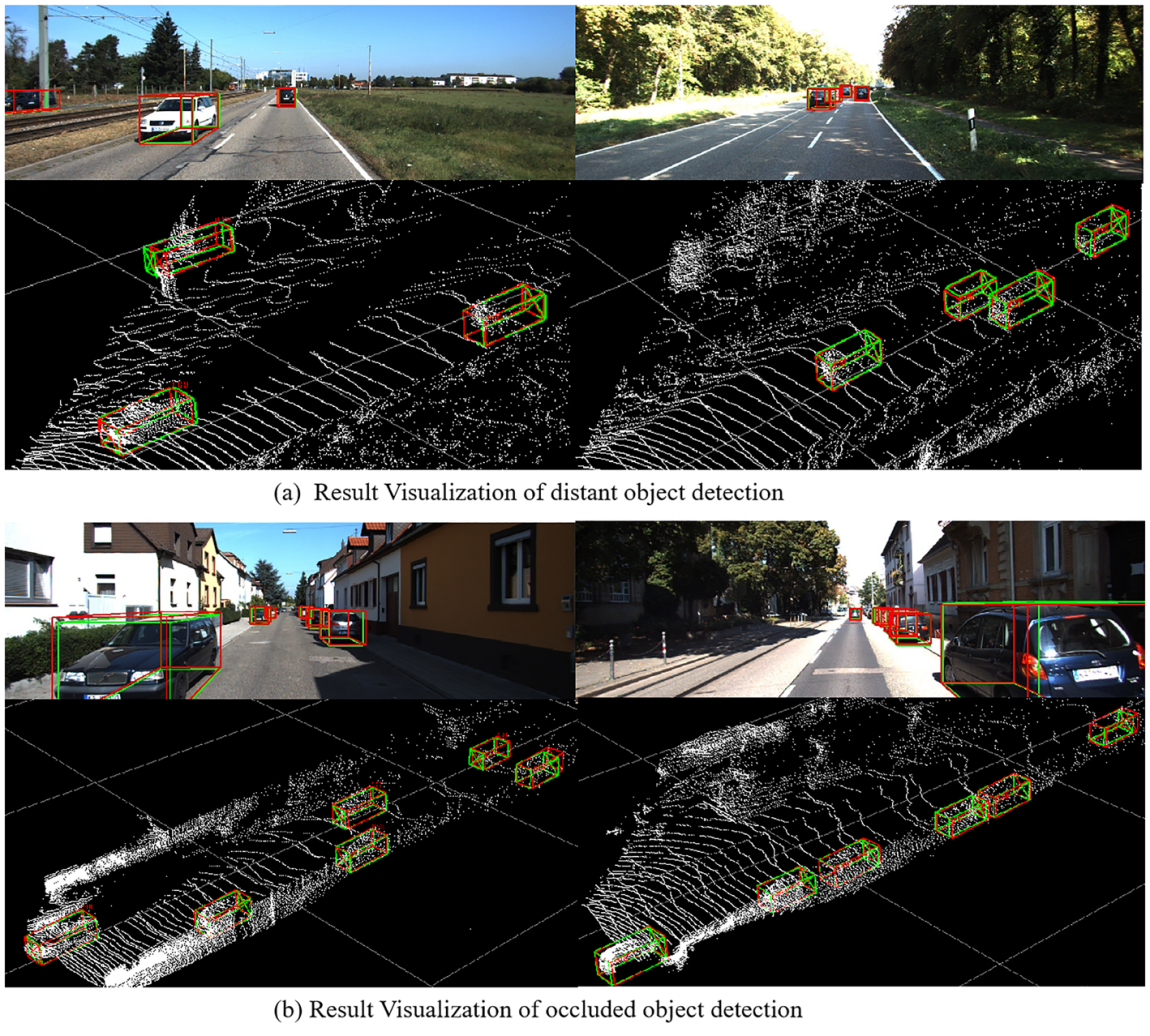
Detection visualization results of distant and occluded vehicle detection.

In addition, in order to obtain an intuitive understanding of the detection performance, the prediction results of this method are compared with ground truth, and the visual display is carried out from partially occluded and distant targets respectively. In the validation set, the renderings of the 3D boundary box obtained by the algorithm in the RGB maps and the corresponding point clouds are shown in Fig. 7, where red represents the 3D boundary box (Ground truth) of real label, and the green represents the 3D box predicted by the proposed method.

As shown in Fig. 7, the accuracy of the center point and length, width, and height of 3D bounding box is infinitely close to ground truth. In summary, the detection network based on multi-modal data fusion has good robustness, which can alleviate the problem of missed and false detection of partial occlusion and distant targets that often occured in traffic scenes.

### Evaluation on KITTI object benchmark test dataset

Through submitting to the official test server of KITTI, results on the test set are obtained. And vehicle performance of the proposed method (with 7D colored point cloud) is compared with other existing algorithms, including LiDAR-based (BirdNet^5^, BirdNet+^6^, RT3D^8^, Pointpillars^10^, VoxelNet^16^, SECOND^17^ and SegVoxelNet^18^, and multi-sensor fusion-based ( MV3D^25^, AVOD^26^, MMF^30^, MVAF-Net^29^, Contfuse^38^ and F-PointNet^40^ ). As shown in Table 2, the detailed Average Precison (mAP) and inference time for 3D vehicle detection and 2D BEV detection are reported. Difficulty levels of easy, moderate, and hard are utilized based on definitions from KITTI official website.

**Table 2:** Performance comparison with LIDAR-based and sensor fusion-based methods for vehicle detection on the KITTI benchmark (AP).

Methods	Sensor modality	Time (s)	3D detection (%)			2D BEV detection (%)		
			Easy	Moderate	Hard	Easy	Moderate	Hard
BirdNet^5^	L (BEV)	0.11	40.99	27.26	25.32	–	–	–
BirdNet+^6^	L (BEV)	0.1	70.14	51.85	50.03	81.85	86.43	75.36
RT3D^8^	L (BEV)	0.09	23.74	19.14	18.86	56.44	44.00	42.34
Pointpillars^10^	L (BEV)	0.016	82.58	74.31	68.99	88.35	86.10	79.83
VoxelNet^16^	L (voxel)	0.23	77.47	65.11	57.73	89.35	79.26	77.39
SECOND^17^	L (voxel)	0.04	84.65	75.96	68.71	88.07	79.37	77.95
SegVoxelNet^18^	L (voxel)	0.04	86.04	76.13	70.76	86.62	86.16	78.68
MV3D^25^	L + C	0.36	74.97	63.63	54.00	85.82	76.90	68.94
AVOD^26^	L + C	0.08	76.39	66.47	60.23	86.80	83.79	77.90
MVAF-Net^29^	L + C	0.06	87.87	78.71	75.48	–	–	–
MMF^30^	L + C	0.08	88.40	77.43	70.22	89.49	87.47	79.10
Contfuse^38^	L + C	0.06	82.54	66.22	64.04	88.81	85.83	77.33
F-PointNet^40^	L + C	0.17	82.19	69.79	60.59	88.70	84.00	75.33
Ours	L + C	0.05	89.14	77.85	73.03	91.74	85.26	79.73

L presents the LiDAR sensor, and C presents the Camera sensor (/%).

For 3D vehicle detection, it is evident from Table 2 that proposed method achieves AP values of 89.14% and 77.85% at easy and moderate levels respectively, with an average inference time of approximately 0.05 s, which is outperforming the result attained by most published methods. For the 2D BEV detection task, the results are approximately the same as 3D vehicle detection task.

In comparison to LiDAR-based methods, the proposed method is better than existing methods in 2D BEV detection task but is slightly worse than Pointpillars^10^ at the hard level. Moreover, the performance of this method exceeds VoxelNet^16^ by 2.39%, and SECOND^17^ by 3.67%, which demonstrates that this model performs better with RGB image and point cloud as input.

In comparison to multi-sensor fusion-based methods, in 3D detection task. From Table 2, it can be found that this method outperforms all previous methods except MVAF-Net^29^ at moderate and hard levels. The proposed method outperforms the MV3D^25^ by 14.17%, AVOD^26^ by 12.75%, and Contfuse^38^ by 6.6% at the easy level, respectively. For Contfuse uses the 3D points to project features from the image to 3D space, the “feature blurring” occurs because feature vector from BEV corresponds to multiple pixels in the image view. For 2D BEV detection task, this method outperforms all the previous methods except MMF^30^ at the moderate level.

The reason for the inferior performance is that MMF^30^ and MVAF-Net^29^ are two-stage methods based on bounding boxes. MMF^30^ and MVAF-Net^29^ adopt multi-view fusion, which not only utilize different projected views of point cloud, but also fuse them in multiple stages. To be more specific, MMF^30^ improves the performance of 3D detection through additional auxiliary tasks (including 2D object detection, ground estimation, and depth complementation), which adds additional labeling efforts.

Although both methods have higher detection performance, they require an additional bounding box optimization process, a more complicated network structure, and a large amount of calculation, which increase running time. Notably, the proposed method does not involve complex subsequent processing (such as IoU-based non-maximal suppression) to filter the overlapping results.

Table 2 also lists the running time of this method with other above-mentioned methods. It can be noticed that this method takes approximately 0.05 s for inference, faster than multi-sensor fusion-based methods, in which the average computing speed on KITTI object detection data set can reach 19.6 FPS. Among the LiDAR-based methods, SECOND^17^ and SegVoxelNet^18^ take 0.04 s for inference, with the accuracy rate in Fourth and Fifth place, respectively.

**Figure 8:**
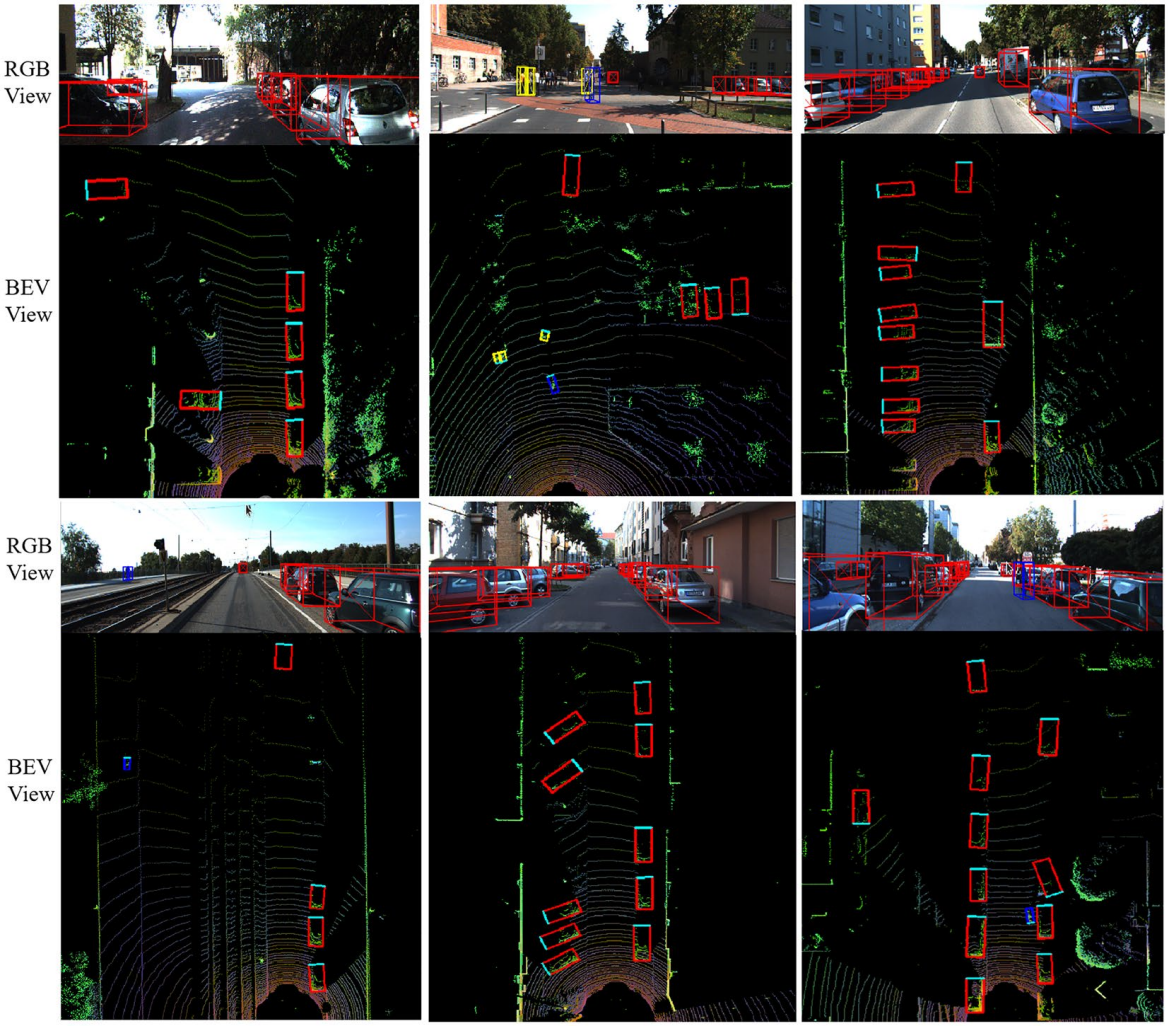
Visualization of detection results. Notice that predictions are entirely based on BEV maps derived from point clouds. Re-projecting to image space is for illustrative purposes only.

For a more intuitive analysis, Fig. 8 represents some predicted visualization results at RGB view and BEV view on the KITTI object detection test set. Pedestrians are indicated by yellow detection boxes, vehicles are represented by red detection boxes, cyclists are represented by blue detection boxes, and the green boundary is the corresponding orientation. Each sub-block is composed of RGB image and its corresponding LiDAR data. Note that the visualization of the prediction results is based on bird's-eye view generated from 7D colored point cloud and re-projected back onto the image for illustrative purposes only.

**Figure 9:**
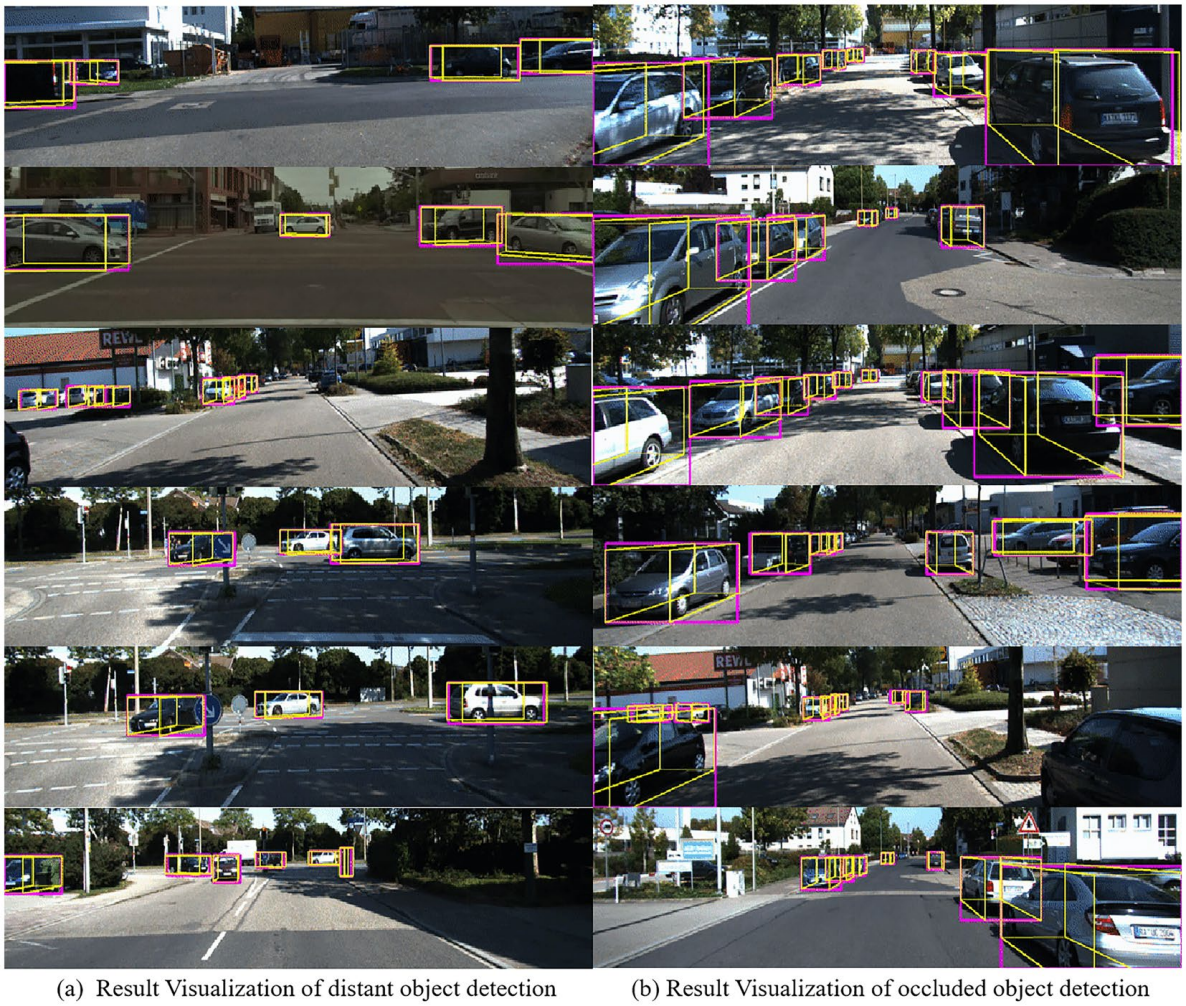
Visualization of detection results on Odometry sequence 05 dataset. 3D detection results (yellow) and 2D detection results (pink) in image space. The left is the sample results on the distant object, and the left is occluded object.

On KITTI Odometry sequence 05 dataset. Figure 9 shows some visualization results in RGB image of proposed method. Specifically, the visualization results of distant are on the left, and the occluded detection results are on the right. Pink represents 2D detection and the yellow represents 3D detection. It can be observed that under different illumination, this algorithm attains good detection results for vehicles. At the same time, the proposed method can better detect vehicle in presence of large traffic volume and partial occlusion. The quantitative experiment on KITTI Odometry Dataset can be shown in Table 3, including 3D vehicle detection and 2D detection.

**Table 3:** Performance for vehicle detection on the KITTI Odometry Dataset ( /%).

	Vehicle			Pedestrian			Cyclist		
	Easy	Moderate	Hard	Easy	Moderate	Hard	Easy	Moderate	Hard
3D detection	74.97	63.63	54.00	52.74	50.11	48.32	61.23	58.03	54.37
2D detection	85.82	76.90	68.94	54.11	51.98	49.44	70.51	59.60	55.59

### Evaluation on nuScenes dataset

**Figure 10:**
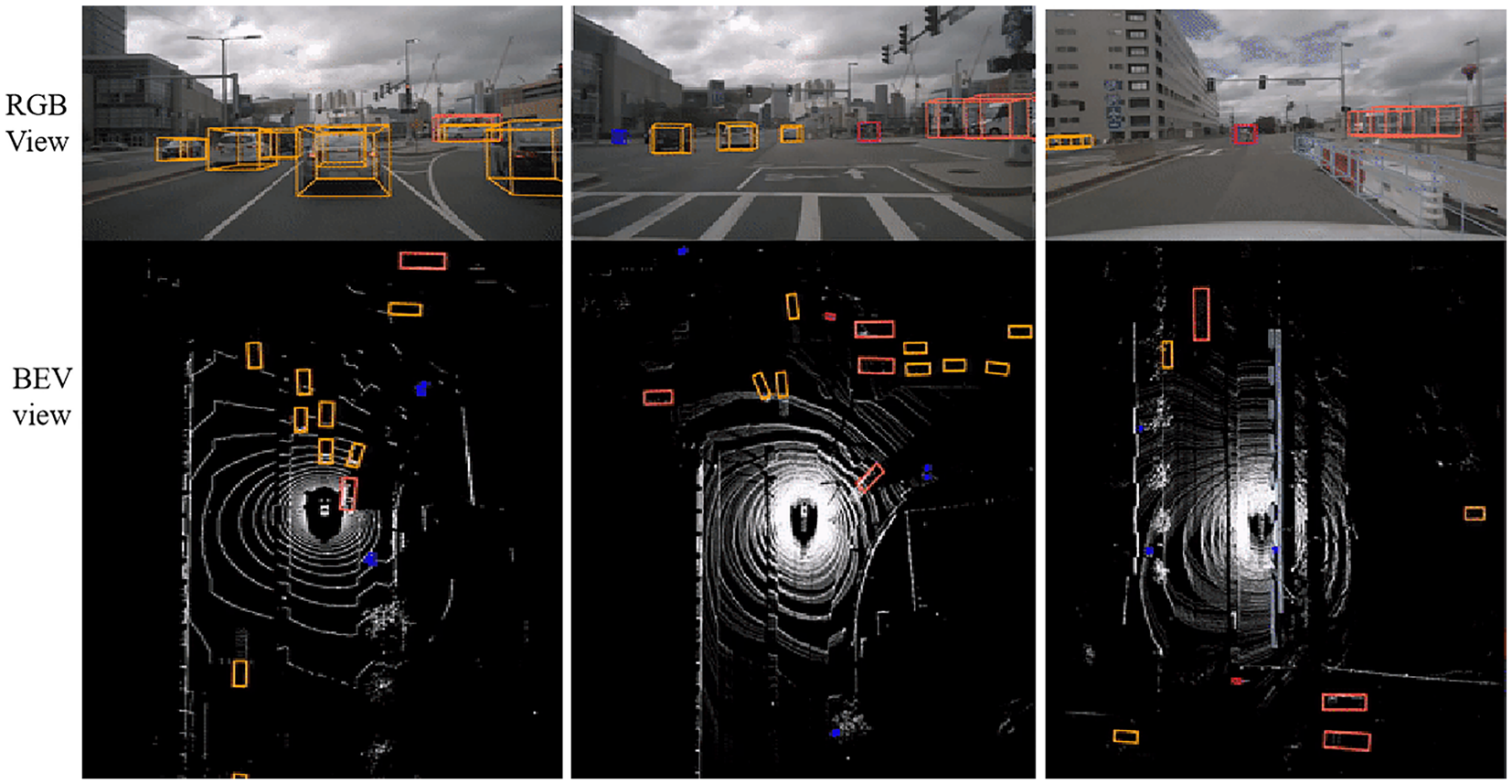
Visualization of detection results on the nuScenes.

We further compare this method with some related methods on nuScenes dataset, which is a large-scale outdoor dataset. As can be shown in Table 4, the best results are in bold. Compared to pointpillars^10^, this method has received great increase in mAP, some categories such as traffic corns, pedestrian and bicycle often have few LiDAR points on them, thus the additional appearance features provided by color and texture information is extremely valuable. The reason that our method having a slightly lower performance than TransFusion^33^ is that the soft-association mechanism to point clouds and image pixels is utilized, and the attention mechanism of TransFusion is adaptively determined where and what information should be taken from image. Some visualization results are shown in Fig. 10, the yellow presents car, the orange presents truck, and blue presents pedestrian.

**Table 4:** Performance comparisons on the nuScene test set (mAP, /%).

Methods	mAP	Car	Truck	Bus	Trailer	Ctr.Vh	Ped	Motor-cycle	Bicycle	Tr.Con	Barrier
Point-pillars^10^	30.5	68.4	23.0	28.2	23.4	4.1	59.7	27.4	1.1	30.8	38.9
Point-painting^22^	46.4	77.9	35.8	36.1	37.3	15.8	73.3	41.5	24.1	62.4	60.2
Trans-Fusion^33^	**68.9**	**87.1**	**60.0**	**68.3**	**60.8**	**33.1**	**88.4**	**73.6**	**52.9**	**86.7**	**78.1**
Ours	55.8	75.71	33.85	38.21	28.54	18.66	65.91	39.69	25.01	50.22	46.55

The results are directly quoted from their original papers.

*Ctr.Vh* construction vehicle, *Ped.* pedestrian, *Tr.Con* traffic cone.

Best values are in bold.

Owing to the sparseness of 3D data, the number of scanned points for distant objects is very small, failing to extract features from enough data for recognition. This method adds color features to the 3D raw point cloud data, so that objects with different colors are distinguishable significantly from the surrounding background environment, thus features can be extracted for detection, and the recognition ability of the algorithm for difficult-to-detect objects is improved.

Under the condition of low visibility and light sensitivity, image features cannot be extracted, which will produce undesirable results. This method is essentially calculated on the point cloud, it can still extract information about the surrounding environment and realize the perception of surrounding environment in event of camera sensor fails.

There are some issues to be considered for the future: first, this paper considers two modalities, when adding the third sensor data, the data distribution from different viewpoints may be different, in this case, accurate calibration is demanded in advance. Second, the network relies on the update of the 2D detector, and results are limited by the existing 2D detection network. At last, the early fusion strategy has been taken into consideration, and the combination form of various fusion strategies should be considered in the future.

## Conclusion

This paper proposes a 3D vehicle detection network based on image and point cloud, which introduces an early fusion module, BEV encoding format, and Feature Fusion (2F) network. Unlike other fusion-based methods, through calibration parameters, image pixels are projected into corresponding LiDAR data in 3D space to generate 7D colored point clouds, and then the obtained 7D colored point clouds are converted to top view for feature extraction. The proposed method can be evaluated by end-to-end training. Experimental results demonstrate that it has better detection performance for occluded and distant vehicles.

## Data Availability

The datasets generated during the current study are available from the corresponding author on reasonable request.

## References

[1] Wang L, Huang Y (2021). A survey of 3D point cloud and deep learning-based approaches for scene understanding in autonomous driving. IEEE Intell. Transp. Syst. Mag..

[2] Li, B., Zhang, T. & Xia, T. Vehicle detection from 3D LiDAR using fully convolutional network. in *Robotics*. arXiv:1608.07916 (Science and Systems Foundation, 2016).

[3] Minemura, K., Liau, H., Monrroy, A. & Kato, S. LMNet: Real-time multiclass object detection on CPU using 3D LiDAR*. *in* Proceedings of the 3rd Asia-Pacific Conference on Intelligent Robot Systems (ACIRS)*. 28–34 (IEEE, 2018).

[4] Zhou, J., Tan, X., Shao, Z. & Ma, L. FVNet: 3D front-view proposal generation for real-time object detection from point clouds. in *Proceedings of the 12th International Congress on Image and Signal Processing, BioMedical Engineering and Informatics (CISP-BMEI)*. 1–8 (2019).

[5] Beltŕan, J. Guindel, C., Moreno, F.M., Cruzado, D., Garćıa, F. & De La Escalera, A. Birdnet: A 3D object detection framework from LiDAR information. in *Proceedings of the IEEE Conference on Intelligent Transportation Systems Conference (ITSC)*. 3517–3523 (2018).

[6] . Barrera, A., Guindel, C., Beltran, J. & García, F. BirdNet+: End to-end 3D object detection in LiDAR bird’s eye view. in *Proceedings of the IEEE Conference on Intelligent Transportation Systems Conference (ITSC).* 1–6 (2020).

[7] Simon, M. *et al*. Complex-yolo: Real-time 3D object detection on point clouds. arXiv preprint arXiv:1803.06199 (2018).

[8] Zeng Y, Hu Y, Liu S, Ye J, Han Y, Li X, Sun N (2018). Rt3d: Real-time 3-D vehicle detection in LiDAR point cloud for autonomous driving. IEEE Robot. Auto Lett..

[9] Yang, B., Luo, W. & Urtasun, R. PIXOR: Real-time 3D object detection from point clouds. in *Proceedings of the IEEE Conference on Computer Vision and Pattern Recognition* (CVPR). 7652–7660 (2018).

[10] Lang, A.H., Vora, S., Caesar, H., Zhou, L., Yang, J. & Beijbom, O. Pointpillars: Fast encoders for object detection from point clouds. in *Proceedings of the IEEE Conference on Computer Vision and Pattern Recognition* (CVPR). 12697–12705 (2019).

[11] . Yang, B., Liang, M. & Urtasun, R. HDNET: Exploiting HD maps for 3D object detection. in *Proceedings of the 2nd Annual Conference on Robot Learning*. 146–155 (2018).

[12] Shi, S., Guo, C., Jiang, L., Wang, Z., Shi, J., Wang, X. & Li, H. PVRCNN: Point-voxel feature set abstraction for 3D object detection, in *Proceedings of the IEEE Conference on Computer Vision and Pattern Recognition* (CVPR). 10526–10535 (2020).

[13] Graham, B. & van der Maaten, L. *Submanifold Sparse Convolutional Networks*. arXiv:1706.01307 (2017).

[14] Graham, B. Spatially sparse convolutional neural networks. in *Proceedings of the IEEE Conference on Computer Vision and Pattern Recognition* (CVPR). arXiv:1409.6070.

[15] Li, B. 3D fully convolutional network for vehicle detection in point cloud. in *Proceedings of the International Conference on Intelligent Robots and Systems* (IROS). 1513–1518 (2017).

[16] Zhou, Y. & Tuzel, O. VoxelNet: End-to-end learning for point cloud based 3D object detection. in *Proceedings of the IEEE Conference on Computer Vision and Pattern Recognition* (CVPR). 4490–4499 (2018).

[17] Yan Y, Mao Y, Li B (2018). SECOND: Sparsely embedded convolutional detection. Sensors.

[18] Yi, S. *et al*. SegVoxelNet: Exploring semantic context and depth aware features for 3D vehicle detection from point cloud. in *Proceedings of the International Conference on Robotics and Automation* (ICRA). 2274–2280 (2020)*.*

[19] Charles, R.Q. *et al*. PointNet: Deep learning on point sets for 3D classification and segmentation. in *Proceedings of the IEEE Conference on Computer Vision and Pattern Recognition* (CVPR). 77–85 (2017).

[20] Yang, Z., Sun, Y., Liu, S., Shen, X. & Jia, J. STD: Sparse-to-dense 3D object detector for point cloud. in *Proceedings of the IEEE International Conference on Computer Vision* (ICCV). 1951–1960 (2019).

[21] Shi, S., Wang, X. & Li, H. PointRCNN: 3D object proposal generation and detection from point cloud. in *Proceedings of the IEEE Conference on Computer Vision and Pattern Recognition* (CVPR). 770–779 (2019).

[22] Vora, S., Lang, A.H., Helou, B. *et al*. PointPainting: Sequential fusion for 3D object detection. in *Proceedings of the IEEE Conference on Computer Vision and Pattern Recognition (CVPR).* 4604–4612 (2020).

[23] Wen LH, Jo KH (2021). Fast and accurate 3D object detection for lidar-camera-based autonomous vehicles using one shared voxel-based backbone. IEEE Access.

[24] Wang, C., Ma, C., Zhu, M. & Yang, X. Pointaugmenting: Cross-modal augmentation for 3D object detection. in *Proceedings of the IEEE/CVF Conference on Computer Vision and Pattern Recognition* (CVPR). 11794–11803 (2021).

[25] Chen, X., Ma, H., Wan, J., Li, B. & Xia, T. Multi-view 3D object detection network for autonomous driving. in *Proceedings of the IEEE/CVF Conference on Computer Vision and Pattern Recognition* (CVPR). 1907–1915 (2017).

[26] Ku, J., Mozifian, M., Lee, J., Harakeh, A., & Waslander, S.L. Joint 3D proposal generation and object detection from view aggregation, in *Proceedings of the IEEE Conference on Intelligent Robots and Systems* (IROS). 1–8 (2018).

[27] Yoo, J. H. *et al*. 3D-CVF: Generating joint camera and LiDAR features using cross-view spatial feature fusion for 3D object detection. in *Proceedings of the European Conference on Computer Vision* (ECCV*)*. 720–736 (2020).

[28] Raffiee, A., & Irshad, H. *Class-Specific Anchoring Proposal for 3D Object Recognition in LIDAR and RGB Images*. arXiv:1907.09081 (2019).

[29] Wang, G., Tian, B., Zhang, Y., Chen, L., Cao, D. & Wu, J. *Multi-View Adaptive Fusion Network for 3D Object Detection*. arXiv: abs/2011.00652 (2020).

[30] Liang, M., Yang, B., Chen, Y., Hu, R. & Urtasun, R. Multi-task multi-sensor fusion for 3D object detection, in *Proceedings of the IEEE/CVF Conference on Computer Vision and Pattern Recognition* (CVPR)*.* 7345–7353 (2019).

[31] Liu, Z., Tang, H., Amini, A., Yang, X., Mao, H., Rus, D. & Han, S. Bevfusion: Multi-task multi-sensor fusion with unified bird’s-eye view representation, *arXiv preprint* arXiv:2205.13542 (2022).

[32] Liang, T., Xie, H., Yu, K., Xia, Z., Lin, Z., Wang, Y., Tang, T., Wang, B. & Tang, Z. Bevfusion: A simple and robust lidar-camera fusion framework. *arXiv preprint* arXiv:2205.13790 (2022).

[33] Bai, X., Hu, Z., Zhu, X., Huang, Q., Chen, Y., Fu, H. & Tai, C.-L. Transfusion: Robust lidar-camera fusion for 3D object detection with transformers. in *Proceedings of the IEEE/CVF Conference on Computer Vision and Pattern Recognition* (CVPR). 1090–1099 (2022).

[34] Jiao, Y., Jie, Z., Chen, S., Chen, J., Wei, X., Ma, L. & Jiang, Y.-G. Msmdfusion: Fusing lidar and camera at multiple scales with multidepth seeds for 3D object detection. *arXiv preprint* arXiv:2209.03102 (2022).

[35] Liu H, Liao K, Lin C, Zhao Y, Guo Y (2020). Pseudo-LiDAR point cloud interpolation based on 3D motion representation and spatial supervision. IEEE Trans. Intell. Transp. Syst..

[36] Liang, M., Yang, B., Chen, Y., Hu, R., & Urtasun, R. Multi-task multi-sensor fusion for 3D object detection. in *Proceedings of the IEEE/CVF Conference on Computer Vision and Pattern Recognition* (*CVPR*), *Long Beach, CA, USA*, *15–20 June 2019*. 7345–7353 (2019).

[37] Gu, S., Yang, J., & Kong, H. A cascaded lidar-camera fusion network for road detection. in *Proceedings of the International Conference on Robotics and Automation* (ICRA). 13308–13314 (IEEE, 2021).

[38] Liang, M., Yang, B., Wang, S. & Urtasun, R. Deep continuous fusion for multi-sensor 3D object detection. in *Proceedings of the European Conference on Computer Vision* (ECCV*)*. 641–656 (2018).

[39] Wang, Z., Zhan, W., & Tomizuka, M. Fusing bird’s eye view lidar point cloud and front view camera image for 3D object detection. in *Proceedings of the IEEE Intelligent Vehicles Symposium (IV)*. 1–6 (IEEE, 2018).

[40] Qi, C.R., Liu, W., Wu, C., Su, H. & Guibas, L.J. Frustum PointNets for 3D object detection from RGB-D data. in *Proceedings of the**IEEE Conference on Computer Vision and Pattern Recognition* (CVPR). 918–927 (2018).

[41] Wang, Z. & Jia, K. Frustum ConvNet: Sliding frustums to aggregate local point-wise features for amodal 3D object detection. in *Proceedings of the International Conference on Intelligent Robots and Systems* (IROS). 1742–1749 (2019).

[42] A Frustum-based probabilistic framework for 3D object detection by fusion of LiDAR and camera data ISPRS. Journal of Photogrammetry and Remote Sensing. 15990–159100. 10.1016/j.isprsjprs.2019.10.015 (2020).

[43] Geiger A, Lenz P, Stiller C, Urtasun R (2013). Vision meets robotics: The KITTI dataset. Int. J. Robot. Res..

[44] He, K., Zhang, X., Ren, S. & Sun, J. Deep residual learning for image recognition. in *Proceedings of the**IEEE Conference on Computer Vision and Pattern Recognition* (CVPR). 770–778 (2016).

[45] Lin, T.-Y. *et al*. Feature pyramid networks for object detection. in *Proceedings of the IEEE Conference on Computer Vision and Pattern Recognition* (CVPR). 2117–2125 (2017).

[46] Caesar, H., Bankiti, V., Lang, A.H., Vora, S., Liong, V.E., Xu, Q., Krishnan, A., Pan, Y., Baldan, G., & Beijbom, O. nuScenes: A multimodal dataset for autonomous driving. in Proceedings of the *IEEE Conference on Computer Vision and Pattern Recognition* (CVPR) (2020).

